# Editorial: Resistance and virulence in Enterobacteriales from different sources

**DOI:** 10.3389/fcimb.2023.1140567

**Published:** 2023-02-07

**Authors:** Gerson Nakazato, Renata Katsuko Takayama Kobayashi, Marcia Regina Eches Perugini, Nilton Lincopan, Eliana Carolina Vespero

**Affiliations:** ^1^Department of Microbiology, Center of Biological Sciences, State University of Londrina, Paraná, Brazil; ^2^Department of Pathology, Clinical and Toxicological Analysis, Center for Health Sciences, State University of Londrina, Paraná, Brazil; ^3^Department of Microbiology, Institute of Biomedical Sciences, University of São Paulo, São Paulo, Brazil

**Keywords:** antimicrobials, pathogenicity, livestock, detection, molecular

The “One Health” concept, firstly named “One Medicine”, has been approached in several studies including antimicrobial resistance and epidemiology and monitoring of multidrug-resistant microorganisms. One Health integrates human health with animals and the environment under different features such as transmission, diagnosis, and prevention. During the COVID-19 pandemic period, this relationship was verified, and information was important for the understanding of this disease and infection prevention.

Bacterial resistance is a considerable threat to public health worldwide. The World Health Organization (WHO) estimated that the number of deaths in the world will be 10 million each year by 2050. Some bacterial isolates showed resistance to all conventional antimicrobials available.

Acquisition of resistance genes and mutations are natural processes; however, these mechanisms can be accelerated by the intensive use of antimicrobials, inadequate surveillance, and the inappropriately controlled regulation of drug use in hospitals even in the farming industry.

Several studies have focused on virulence and resistance genes together in mobile genetic elements, which are associated with the dissemination of genes and microbial adaptation to different environments.

In this Research Topic, five articles approached the spreading of bacterial virulence and resistance among Enterobacteriales from different sources and their impacts on animals and environments.

The epidemiology of infections caused by multidrug-resistant bacteria in hospitals is very evident, suggesting important monitoring of the spread of these microorganisms. Rodriguez et al. showed the AmpC beta-lactamase prevalence among *Escherichia coli* and *Klebsiella pneumoniae* isolates from urine samples at the University Hospital of the West Indies Jamaica by using molecular techniques (whole-genome sequencing). The results of this study suggested the requirement of using molecular methods compared to the disc approximation test.

In another study about polymyxin resistance in clinical isolates of *K. pneumoniae* carbapenemase (KPC)-producing bacteria from eight states of Brazil, Conceição-Neto et al. showed that clonality studies and missense mutations in chromosomal genes are very important for understanding the clonal dissemination and relationship of these superbugs.

Continuing on genetic and molecular studies, although with a virulence approach, Fan et al. reported a novel hybrid plasmid in an ST23 hypervirulent *K. pneumoniae* showing passage instability during the conjugation process. The authors suggested that continuous monitoring of the acquisition of conjugative virulence plasmids may have critical value for studies on these genetic elements.

Studies about virulence approaches depend on different features such as source, host immunology, and genetic and pathogen types. Two studies in our Research Topic focused on *Salmonella enterica*, which is an important human pathogen of foodborne infections.

Song et al. demonstrated that a critical factor of the Type VI Secretion System (TSS6) in *Salmonella* Enteritidis contributes to the invasion process of these bacteria, induction of duck granulosa cells, and trigger of immune responses increasing egg contamination. The authors concluded that the findings of this study offer a novel candidate for the development of new vaccines against *S.* Enteritidis.

Fratty et al. studied the expression of one of the proteins in the *bcs* complex enhanced with the survival of *Salmonella* Typhimurium on parsley. The authors verified that the overexpression of BcsZ improved the epiphytic and endophytic survival of *S.* Typhimurium in an “in-planta” model, suggesting the importance of this protein in the *Salmonella*–plant interactions.

The articles of our Research Topic provided a notion about the relevance of resistance to antimicrobials and virulence in Enterobacteriales, even as their sources with “One Health” approach (human, environment, and animals) showing different features (genetic, immunology, molecular biology, and host–pathogen interactions).

New perspectives are expected in future studies about this matter in Enterobacteriales, once these bacteria emerge and are of concern to public health worldwide.

## Author contributions

All authors listed have made a substantial, direct, and intellectual contribution to the work and approved it for publication.

